# Ribosomal Protein Rps26 Influences 80S Ribosome Assembly in *Saccharomyces cerevisiae*

**DOI:** 10.1128/mSphere.00109-15

**Published:** 2016-02-24

**Authors:** Alexander Belyy, Nadezhda Levanova, Irina Tabakova, Sabine Rospert, Yury Belyi

**Affiliations:** a Department of Bacterial Infections, Gamaleya Research Center, Moscow, Russia; b Faculty of Biology, Lomonosov Moscow State University, Moscow, Russia; c Institute of Biochemistry and Molecular Biology, Albert Ludwig University of Freiburg, Freiburg, Germany; d BIOSS Centre for Biological Signaling Studies, University of Freiburg, Freiburg, Germany; e FRIAS Freiburg Institute for Advanced Studies, Albert Ludwig University of Freiburg, Freiburg, Germany; Carnegie Mellon University

**Keywords:** 40S subunit, *Saccharomyces cerevisiae*, eukaryote-specific motif, mutagenesis, ribosomal protein, ribosome assembly, translation initiation, yeast genetics

## Abstract

The eukaryotic ribosome consists of a small (40S) and a large (60S) subunit. Rps26 is one of the essential ribosomal proteins of the 40S subunit and is encoded by two almost identical genes, *RPS26a* and *RPS26b*. Previous studies demonstrated that Rps26 interacts with the 5′ untranslated region of mRNA via the eukaryote-specific 62-YXXPKXYXK-70 (Y62–K70) motif. Those observations suggested that this peptide within Rps26 might play an important and specific role during translation initiation. By using alanine-scanning mutagenesis and engineered strains of the yeast *Saccharomyces cerevisiae*, we found that single amino acid substitutions within the Y62–K70 motif of Rps26 did not affect the *in vivo* function of the protein. In contrast, complete deletion of the Y62–K70 segment was lethal. The simultaneous replacement of five conserved residues within the Y62–K70 segment by alanines resulted in growth defects under stress conditions and produced distinct changes in polysome profiles that were indicative of the accumulation of free 60S subunits. Human Rps26 (Rps26-Hs), which displays significant homology with yeast Rps26, supported the growth of an *S. cerevisiae* Δ*rps26a* Δ*rps26b* strain. However, the Δ*rps26a* Δ*rps26b* double deletion strain expressing Rps26-Hs displayed substantial growth defects and an altered ratio of 40S/60S ribosomal subunits. The combined data strongly suggest that the eukaryote-specific motif within Rps26 does not play a specific role in translation initiation. Rather, the data indicate that Rps26 as a whole is necessary for proper assembly of the 40S subunit and the 80S ribosome in yeast.

**IMPORTANCE** Rps26 is an essential protein of the eukaryotic small ribosomal subunit. Previous experiments demonstrated an interaction between the eukaryote-specific Y62–K70 segment of Rps26 and the 5′ untranslated region of mRNA. The data suggested a specific role of the Y62–K70 motif during translation initiation. Here, we report that single-site substitutions within the Y62–K70 peptide did not affect the growth of engineered yeast strains, arguing against its having a critical role during translation initiation via specific interactions with the 5′ untranslated region of mRNA molecules. Only the simultaneous replacement of five conserved residues within the Y62–K70 fragment or the replacement of the yeast protein with the human homolog resulted in growth defects and caused significant changes in polysome profiles. The results expand our knowledge of ribosomal protein function and suggest a role of Rps26 during ribosome assembly in yeast.

## Abstract (precis)

Rps26 is an essential protein of the eukaryotic small ribosomal subunit. Previous experiments demonstrated an interaction between the eukaryote-specific Y62–K70 segment of Rps26 and the 5′ untranslated region of mRNA. The data suggested a specific role of the Y62–K70 motif during translation initiation. Here, we report that single-site substitutions within the Y62–K70 peptide did not affect the growth of engineered yeast strains, arguing against its having a critical role during translation initiation via specific interactions with the 5′ untranslated region of mRNA molecules. Only the simultaneous replacement of five conserved residues within the Y62–K70 fragment or the replacement of the yeast protein with the human homolog resulted in growth defects and caused significant changes in polysome profiles. The results expand our knowledge of ribosomal protein function and suggest a role of Rps26 during ribosome assembly in yeast.

## INTRODUCTION

The ribosome represents an essential component of the translational machinery in prokaryotes, archaea, and eukaryotic organisms (1). In the yeast *Saccharomyces cerevisiae*, the ribosome consists of two subunits (small 40S and large 60S), which contain 79 ribosomal proteins. Fifty-nine ribosomal proteins are encoded by duplicated genes. Twenty-one duplicated genes are translated into identical polypeptides, while the remainder are translated into very similar proteins (2). The majority of ribosomal proteins are essential for yeast growth (3). Most ribosomal proteins are quite conserved through different kingdoms of life. For instance, 35 ribosomal proteins possess homologs in eukarya, archaea, and eubacteria. Eukarya and archaea additionally share 33 ribosomal proteins, while only 12 proteins are specific for eukaryotic ribosomes (1, 4).

Rps26 was originally isolated from rat liver in 1977 (5). The corresponding mammalian gene was cloned from hamster and human cDNAs (6, 7). Subsequently, Rps26 was expressed in *Escherichia coli*, and the purified protein was shown to suppress splicing of its own pre-mRNA (8, 9). This observation suggested the existence of some feedback mechanism controlling Rps26 synthesis; however, the functional significance of this phenomenon is still unclear. Rps26 has no obvious eubacterial counterpart; however, the eubacterial ribosomal protein S18 contains a similar rRNA-contacting structural motif and was therefore suggested to be the functional homolog of Rps26 (10).

Yeast cells contain two copies of *RPS26*, *RPS26a* (located on chromosome 7) and *RPS26b* (located on chromosome 5), which are 92% identical. On the protein level, Rps26a differs from Rps26b by only two residues (E106D and D113A) in the C-terminal domain of the protein. Both proteins consist of 119 amino acids and possess a molecular mass of approximately 14 kDa (http://www.yeastgenome.org/). The Δ*rps26a* Δ*rps26b* double deletion is lethal, indicating that Rps26 is essential for the life of yeast (11).

Interest in Rps26 was aroused recently due to its possible involvement in the pathogenesis of Diamond-Blackfan anemia, an inherited human bone marrow failure syndrome, characterized by the development of anemia during childhood (12). Indeed, numerous studies demonstrated that mutations in several genes encoding ribosomal proteins, including *RPS26a/b*, might be linked to Diamond-Blackfan anemia (13–15). In addition, Rps26 was shown to participate in a variety of cellular processes not directly associated with translation, such as p53 activity, endoplasmic reticulum (ER) stress, the NEDD8 pathway, nonsense-mediated mRNA decay, and filamentous growth (2, 11, 16–18).

**FIG 1:**
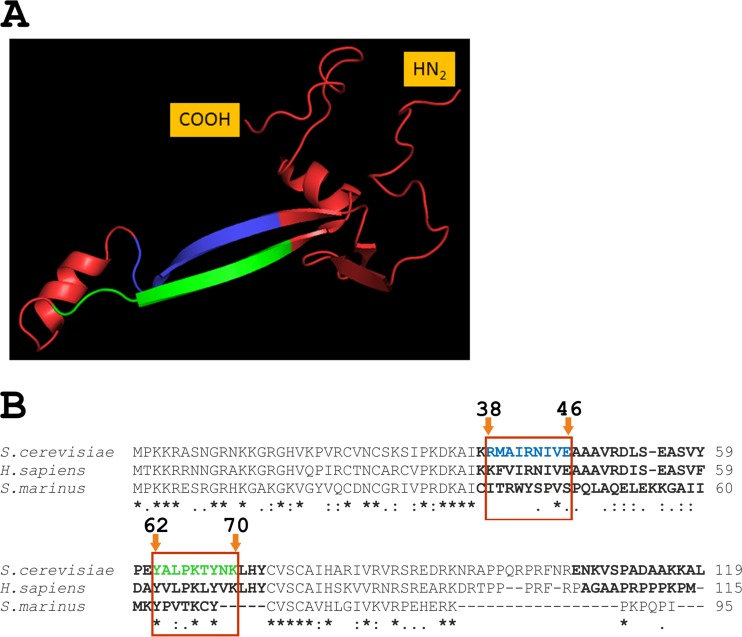
Structural features of Rps26a. (A) Crystal structure of *S. cerevisiae* Rps26 (adapted from PDB 3U5G and drawn using PyMOL Molecular Graphics System [Schrodinger, Germany]). The β-strand containing the eukaryote-specific motif 62-YALPKTYNK-70 (22) and its mirror sequence on the opposite β-strand are shown in green and blue, respectively. The COOH and NH_2_ termini are indicated. (B) Multiple alignment of Rps26 proteins from *S. cerevisiae*, *Homo sapiens*, and *Staphylothermus marinus*. The eukaryote-specific motif and its mirror sequence are shown in green and blue, respectively. Sequence regions of low similarity, located predominantly within both β-strands and in the α-helical region in the middle of the molecule, are indicated in boldface. The amino acid numbers are based on the sequence of *S. cerevisiae* Rps26a.

Rps26 is located within the small ribosomal subunit in close proximity to Rps1, Rps5, Rps14, and Rps28 (19). Elegant *in vitro* studies, using artificial mRNA molecules with uniquely positioned photoactivated nucleotide analogs, demonstrated that Rps26 was cross-linked to nucleotides within mRNA molecules positioned from −4 to −9 relative to nucleotide +1 located in the ribosomal P site (20, 21). Another study on the topic revealed that the contact between the mRNA and Rps26 was established via a short segment (62-YXXPKXYXK-70; termed the Y62–K70 motif below) located in an antiparallel β-sheet of Rps26 (Fig. 1A) (22). Because the Y62–K70 segment is highly conserved in eukaryotic Rps26 but not in the archaeal homologs, the segment was termed “eukaryote-specific motif” of Rps26 (Fig. 1B) (22). Based on the available crystal structures of ribosome complexes, specific interactions of the Y62–K70 segment with the translated mRNA molecules were identified (23, 24). In particular, it was suggested that lysines K66 and/or K70 interact with mRNA phosphates, while tyrosines Y62 and/or Y68 might participate in binding to protein(s) involved in translation, e.g., with the translation initiation factor eIF3 (22). Proline P65 was suggested to facilitate bending of the Rps26 polypeptide chain, which can be important for the maintenance of functionally competent protein conformation (22).

In order to gain further insight into the mechanism of Rps26 function and, specifically, into the role of the Y62–K70 segment, we employed yeast as a model organism. Based on published data (22), we expected that specific contacts between the Y62–K70 motif and the mRNA would critically depend on one or more of the residues within the eukaryote-specific segment. However, we found that single-site substitutions of the residues within the Y62–K70 segment did not affect the *in vivo* function of Rps26. Only simultaneous replacement of the highly conserved residues Y62, P65, K66, Y68, and K70 with alanine resulted in moderate growth defects and changes in polysome profiles that were indicative of the accumulation of free 60S subunits. The combined findings of this study point toward an important role for Rps26 in ribosome assembly and subunit joining; however, the findings are inconsistent with the idea of a specific role of the Y62–K70 segment during translation initiation.

## RESULTS

It was previously reported that Rps26 contacts the 5′ untranslated region of mRNA (20, 21) via the Y62–K70 segment (22). Based on these observations, it was suggested that the Y62–K70 motif played an important role in mRNA positioning within the 40S ribosomal subunit during initiation of translation (22). If the hypothesis was correct, the replacement of amino acid residues within the Y62–K70 segment should have a significant effect on translation initiation and negatively affect the growth of yeast strains expressing such a mutant version of Rps26.

To test this hypothesis, we initially engineered a haploid yeast strain in which the lethality of the Δ*rps26a* Δ*rps26b* deletions was rescued by the expression of *RPS26a* from a *URA3*-based plasmid (see Text S1 and Table S1 in the supplemental material). Employing the 5-fluoroorotic acid (5-FOA) shuffling method (25), we then replaced the plasmid encoding wild-type Rps26a with a collection of plasmids encoding mutant versions of Rps26 containing single-amino-acid substitutions within the Y62–K70 segment (Fig. 1).

10.1128/mSphere.00109-15.1 Text S1  Construction of *S. cerevisiae* Δ*rps26a* Δ*rps26b* strains. List of supplemental references. Download Text S1, DOCX file, 0.1 MB. Copyright © 2016 Belyy et al. 2016 Belyy et al. This content is distributed under the terms of the Creative Commons Attribution 4.0 International license.

10.1128/mSphere.00109-15.7 Table S1  Strains used in the current study. Download Table S1, DOCX file, 0.1 MB. Copyright © 2016 Belyy et al. 2016 Belyy et al. This content is distributed under the terms of the Creative Commons Attribution 4.0 International license.

**FIG 2:**
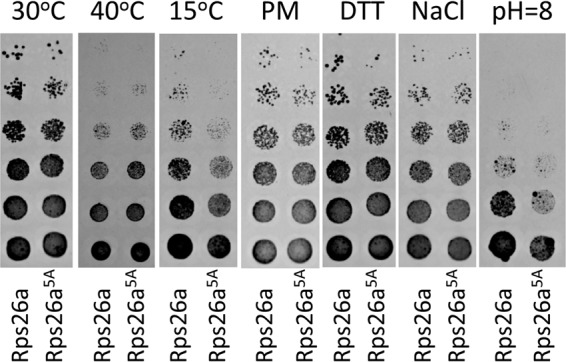
Growth analysis of *S. cerevisiae* strains expressing wild-type Rps26a or Rps26^5A^. Yeast strains were cultivated in YPD at 30°C, 40°C, or 15°C or in YPD plus 100-µg/ml paromomycin (PM), 10 mM DTT, 1 M NaCl, or 50 mM Tris-HCl (pH=8) at 30°C. Plates were incubated for 2 to 5 days depending on growth conditions and supplements.

Surprisingly, the alanine scan through the Y62–K70 segment revealed that all of the engineered Rps26 variants, in which single amino acids were mutated, complemented the growth of the Δ*rps26a* Δ*rps26b* strain, as did the wild-type Rps26 protein (see Fig. S1 in the supplemental material). Even a mutant with the simultaneous replacement of the 5 conserved amino acid residues (Y62, P65, K66, Y68, and K70) by alanine (Rps26a^5A^) complemented the growth of the Δ*rps26a* Δ*rps26b* strain on yeast extract-peptone-dextrose (YPD) medium at 30°C or 40°C in the presence of the translational inhibitor paromomycin, high concentrations of dithiothreitol (DTT), or NaCl. Only at low temperature or if the strains were grown at alkaline pH did the Rps26a^5A^ strain display a moderate growth defect (Fig. 2).

10.1128/mSphere.00109-15.2 Figure S1  Drop test analysis of growth phenotypes of yeast strains expressing Rps26 variants with single alanine substitutions within the 62-YALPKTYNK-70 peptide. (A) Wild-type *RPS26a* was expressed under its own promoter (NativePromo) in strain SC288 (see Table S1 in the supplemental material) or under the control of the *TEF1* promoter (Tef1Promo) in strain SC306. Mutated versions of *RPS26a* (coding for Rpl26a-L64A, -Y62A, -Y68A, -P65A, -K66A, -T67A, -N69A, -K70A, and -L71A in strains SC289, SC290, SC307 to SC310, and SC319 to SC321, respectively; see Table S1) were expressed under the control of the *TEF1* promoter. YPD plates were incubated at 30°C for 3 days. (B) Wild-type (WT) *RPS26a* or versions of *RPS26a* with the alanine substitutions for conserved amino acid residues (shown in boldface) in the 62-**Y**XX**PK**X**Y**X**K**-70 motif were expressed under the control of the *TEF1* promoter. YPD plates were cultivated at 30°C or 15°C for 2 days or 5 days, respectively. Download Figure S1, PDF file, 0.2 MB. Copyright © 2016 Belyy et al. 2016 Belyy et al. This content is distributed under the terms of the Creative Commons Attribution 4.0 International license.

10.1128/mSphere.00109-15.3 Figure S2  Drop test analysis of yeast strains expressing wild-type or C-terminally c*-myc*-tagged Rps26. Wild-type (Rps26) or c*-myc*-tagged Rps26a (Rps26a::*c*-myc) was expressed under the control of the *TEF1* promoter in *S. cerevisiae* (*rps26a*::*LEU2 rps26b*::*TRP1*) (strains SC306 and SC699, respectively; see Table S1). The growth phenotype was studied using drop test analysis on YPD plates at 30°C for 3 days (top). The production of c*-myc*-tagged Rps26 was analyzed by Western blotting with anti-c*-myc* antibodies; anti-Rps9 antibody served as a loading control (bottom). Download Figure S2, PDF file, 0.1 MB. Copyright © 2016 Belyy et al. 2016 Belyy et al. This content is distributed under the terms of the Creative Commons Attribution 4.0 International license.

**FIG 3:**
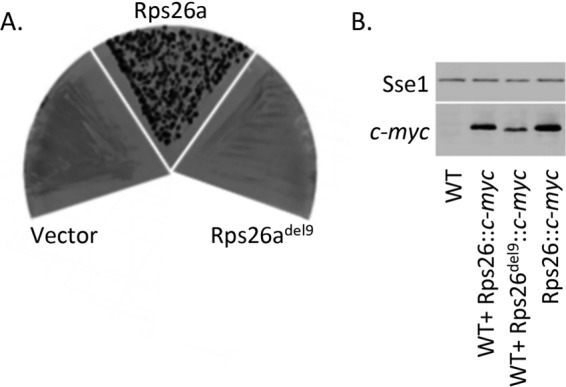
Viability of yeast strains expressing different variants of Rps26. (A) Strains of *S. cerevisiae* Δ*rps26a* Δ*rps26b* carrying pRPS26a (*URA3*) and either pRS313 (vector), pRS313-Rps26a, or pRS313-Rps26a^del9^ (see Table S1) were grown on single 5-FOA plates for 3 to 4 days at 30°C. (B) Total yeast extracts were prepared from wild-type yeast (WT), wild-type yeast expressing c*-myc*-tagged Rps26a (WT+Rps26a::c*-myc*) or c*-myc*-tagged Rps26a^del9^ (WT+Rps26a^del9^::c*-myc*), or Δ*rps26a* Δ*rps26b* yeast expressing c*-myc*-tagged Rps26a (Rps26::c*-myc*). Aliquots were analyzed by Western blotting using anti-c*-myc* antibody and anti-Sse1 antibody as a loading control.

We next tested the effect of deleting the whole Y62–K70 segment (Rps26^del9^) on the functionality of Rps26. The results of the experiment revealed that Rps26^del9^ failed to rescue the lethality of the Δ*rps26a* Δ*rps26b* mutation (Fig. 3A). Because an antibody against Rps26 was not available, Rps26^del9^ was C-terminally fused to the c*-myc* tag. Side-by-side analysis revealed that Rps26^del9^::c*-myc* was expressed, though at lower levels than c*-myc*-tagged Rps26, which served as a wild-type control (Fig. 3B). Thus, most likely Rps26^del9^ was integrated into 40S subunits but was not functional.

**FIG 4:**
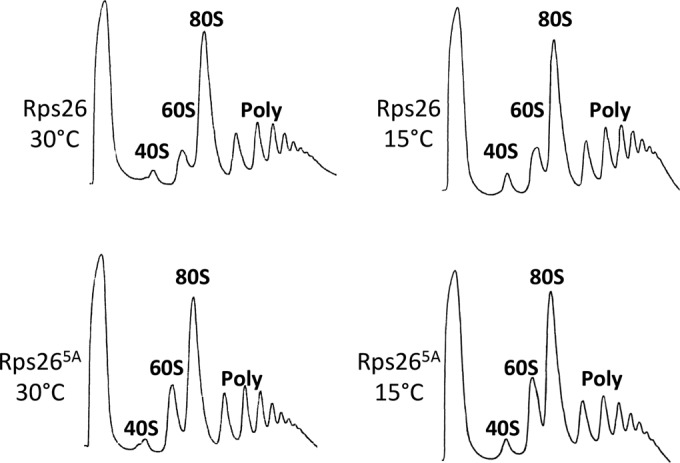
Polysome profile analysis of yeast cells producing Rps26a or Rps26a^5A^ at 30°C or 15°C. *S. cerevisiae* variants and growth temperatures are indicated. Ribosome sedimentation was controlled by monitoring *A*_254_. Peaks showing 40S, 60S, 80S, and polysome (Poly) contents are indicated.

**FIG 5:**
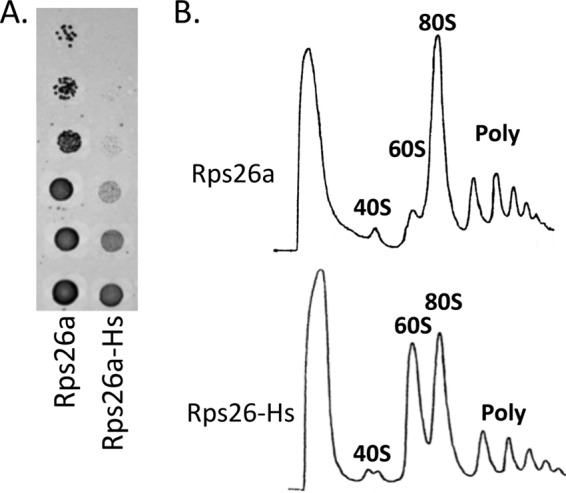
Viability of yeast strains expressing human Rps26-Hs. (A) Serial dilutions of *S. cerevisiae* Δ*rps26a* Δ*rps26b* complemented by plasmid-encoded yeast Rps26a or human Rps26-Hs were spotted onto YPD plates and cultivated for 3 days at 30°C. (B) Yeast Δ*rps26a* Δ*rps26b* complemented with Rps26a or human Rps26-Hs was grown at 30°C to mid-log phase in SDex liquid medium and then analyzed as described in Materials and Methods. Ribosome sedimentation was monitored at 254 nm. Peaks showing 40S, 60S, and 80S subunits and polysome (Poly) contents are indicated.

To examine the reason for the cold-sensitive phenotype of the Rps26^5A^ strain in more detail, we analyzed the ribosome profiles of yeast strains grown at 15°C or 30°C (Fig. 4A to D). A direct comparison revealed that the 60S peak in the ribosome profiles of the Rps26^5A^ strain was increased compared to that in the wild-type strain (Fig. 4). The effect was even more pronounced after growth of the strains at 15°C (Fig. 4). The increased amounts of free 60S subunits in the Rps26^5A^ mutant suggested a functional defect in 40S subunit formation and/or 80S ribosome assembly. To further explore this possibility, we studied human Rps26 (Rps26-Hs), which shares 63% amino acid sequence identity with yeast Rps26 (see the introduction and Fig. 1B). Rps26-Hs supported the growth of the Δ*rps26a* Δ*rps26b* strain; however, the Rps26-Hs strain displayed slow growth even when cultivated on YPD medium at 30°C (Fig. 5A), and extracts prepared from the Rps26-Hs strain showed strong increases of the 60S peak during polysome profile analysis (Fig. 5B).

**FIG 6:**
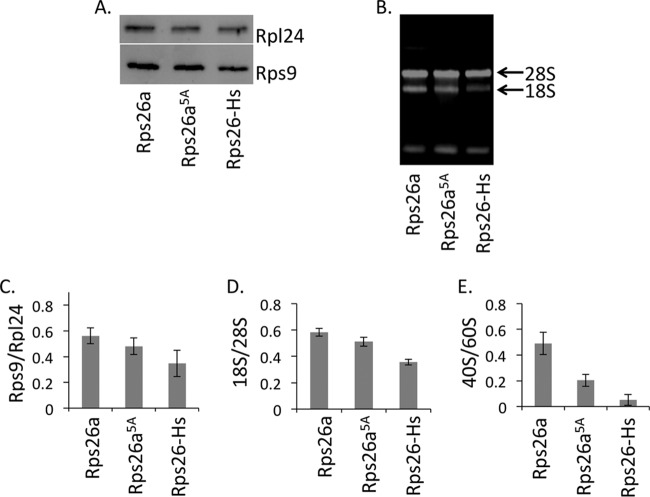
Ratios of 40S/60S subunits in Δ*rps26a* Δ*rps26b* strains complemented by wild-type Rps26a, Rps26a^5A^, or Rps26-Hs. (A) Total extracts were generated as described in Materials and Methods and analyzed by Western blotting with anti-Rps9 (40S ribosomal subunit marker) and anti-Rpl24 (60S ribosomal subunit marker) antisera. The results from one representative experiment are shown. (B) Agarose gel analysis of total rRNA isolated from strains as indicated in panel A. (C) Detected bands shown in panel A were quantified using ImageJ (57). The relative ratios of Rps9/Rpl24 are the means of the results of 2 independent experiments with 3 replicates each. The standard deviations are indicated. The band intensities of Rps9 and Rpl24 were determined in the same lane. The differences between the subunit ratios in Rps26a, Rps26a^5A^, and Rps26-Hs strains are not significant. (D) rRNA bands shown in panel B were quantified using ImageJ (57). The relative ratios of 18S/28S rRNA are the mean results from 2 independent RNA preparations with 4 replicates each. The standard deviations are indicated. The difference between the ratios of 18S/28S rRNA in Rps26a and Rps26a-Hs is significant (*P* < 0.05, *n* = 8). (E) 40S and 60S peaks in polysome profiles of strains grown at 30°C (shown in Fig. 4 and 5) were subjected to quantification via ImageJ software (57). Shown are the relative ratios of the 40S/60S subunits in profiles from Rps26a, Rps26^5A^, and Rps26a-Hs strains. The data represent the mean results from 3 independent experiments. The standard deviations are indicated. Example of profiles are shown in Fig. S3 in the supplemental material.

To further study defects of yeast strains expressing Rps26a^5A^ or Rps26-Hs, we analyzed the ratios between small and large ribosomal subunits. To that end, we compared the expression levels of ribosomal proteins Rps9 (small subunit) and Rpl24 (large subunit) by Western blotting, the amount of 18S rRNA (small subunit) and 28S rRNA (large subunit) by agarose gel electrophoresis, and the area below the 40S and 60S peak curves by polysome profiling (Fig. 6; see also Fig. S3 in the supplemental material).

10.1128/mSphere.00109-15.4 Figure S3  Measurement of 40S and 60S peaks in yeast strains expressing wild-type Rps26, Rps26^5A^ or Rps26-Hs. Areas of 40S and 60S peaks chosen for quantitation (8) are shown in red. Download Figure S3, PDF file, 0.1 MB. Copyright © 2016 Belyy et al. 2016 Belyy et al. This content is distributed under the terms of the Creative Commons Attribution 4.0 International license.

10.1128/mSphere.00109-15.5 Figure S4  PCR analysis of the *S. cerevisiae* deletion strains. (A) Schematic representation of PCRs with the primers used to check marker gene insertions into *RPS26a/b*. Annealing areas and direction of amplifications are shown by colored arrows. (B) Agarose gels showing results of PCRs with the wild-type yeast (WT) and *S. cerevisiae* (*RPS26a/rps26a*::*LEU2 RPS26b/rps26b*::*TRP1*) (strain SC222). Lanes (M) contain molecular mass markers; actual sizes are shown on the left and right sides of the gels. The products of reactions with primer pairs #734/#519, #734/#724, #733/#746, and #733/#521 are shown. The appearance of 1.1-kb and 1.25-kb bands with the marker-specific primer pair #521/#519, as well as the synthesis of 2.37-kb and 2.8-kb bands in addition to the 1.7-kb band for *LEU2* and *TRP1*, respectively, demonstrates correct insertion of the *LEU2/TRP1* markers into the *RPS26* loci. Download Figure S4, PDF file, 0.1 MB. Copyright © 2016 Belyy et al. 2016 Belyy et al. This content is distributed under the terms of the Creative Commons Attribution 4.0 International license.

10.1128/mSphere.00109-15.6 Figure S5  (A) Tetrad analysis of *S. cerevisiae* (*RPS26a/rps26a*::*LEU2 RPS26b/rps26b*::*TRP1*) (strain SC222; see Table S1 in the supplemental material). Spores from 16 tetrads were individually isolated with a dissecting microscope and were allowed to grow for 3 days on YPD plates at 30°C. Note the slow-growth defect of the *rps26a*::*LEU2 RPS26b* strain (9). (B) Marker analysis of haploid yeast colonies obtained from the dissection whose results are shown in panel A. Colonies (A to D) randomly obtained from five tetrads were cultivated on YPD or on minimal SDex medium supplemented with histidine (h), adenine (a), leucine (l), and tryptophane (t) as indicated. The diploid strain SC222 was employed as a positive control. Download Figure S5, PDF file, 0.1 MB. Copyright © 2016 Belyy et al. 2016 Belyy et al. This content is distributed under the terms of the Creative Commons Attribution 4.0 International license.

The minor differences between the ratios of Rps9 and Rpl24 in total extracts obtained from strains expressing either Rps26, Rps26a^5A^, or Rps26-Hs were noticeable but not statistically significant (Fig. 6A and C). Analysis of total 28S and 18S rRNA revealed a small but significant reduction of the total amount of 18S rRNA compared to the amount in the wild-type strain in the Rps26-Hs mutant but not in the Rps26a^5A^ mutant (Fig. 6B and D). Only if the ratio between free 40S and 60S subunits was compared directly via quantification of only the 40S and 60S peaks in the polysome profiles did the shortage of 40S subunits in the Rps26a^5A^ and Rps26-Hs strains become clearly evident (Fig. 6E; see also Fig. S3 in the supplemental material). The data suggested that the moderately increased 60S peak in ribosome profiles of yeast expressing Rps26a^5A^ (Fig. 4) and the strongly increased 60S peak in ribosome profiles of yeast expressing Rps26a-Hs (Fig. 5) resulted from combined defects in subunit joining and 40S subunit deficiency.

## DISCUSSION

Ribosome assembly is assisted by more than 200 assembly factors and 76 small nucleolar RNAs (26). The 40S ribosomal subunit consists of a single 18S rRNA (approximately 1.8 kb) and 33 ribosomal proteins (23). Most of the eukaryote-specific ribosomal proteins and ribosomal proteins with eukaryote-specific extensions, including Rps26, are located on the solvent-exposed surface of the small subunit (23). According to UV-cross-linking experiments, Rps26 specifically interacts with the 5′ untranslated region of mRNA molecules (20, 21). Based on the data (see the introduction and references 20 to 22), it was speculated that Rps26, via the Y62–K70 segment, was directly involved in docking of the mRNA to the 40S subunit during translation initiation.

To test the hypothesis that specific contacts between the Y62–K70 segment of Rps26 and mRNA are functionally important, we utilized genetic experiments in the model organism *S. cerevisiae*. Initially, we replaced each residue within the Y62–K70 segment with alanine. We speculated that if the Y62–K70 segment of Rps26 was indeed necessary for mRNA binding and translation initiation, yeast strains expressing such point mutants of Rps26 should display severe growth defects. Surprisingly, however, none of the point mutations within the Y62–K70 segment caused obvious growth defects in yeast. The finding indicated that strict conservation of the Y62–K70 segment was not critical for the function of Rps26 and argued against a specific role of the residues within the motif. Even the simultaneous replacement of 5 highly conserved amino acid residues within the Y62–K70 peptide in the Rps26^5A^ mutant caused only mild growth defects.

One of the phenotypes of the Rps26^5A^ strain was moderate cold sensitivity. This mutant of Rps26 thus resembles many previously described variants with alterations within ribosomal proteins, which cause ribosome assembly defects (27–31). Deficiency in 80S ribosome assembly is often reflected by anomalously high 40S or 60S peaks in polysome profiles (32, 33). Our results obtained with the Rps26-Hs and Rps26^5A^ strains suggested that proper ribosome assembly required functional Rps26.

The recently solved crystal structure of the 40S ribosomal subunit revealed a dumbbell-like fold of the yeast protein, in which peripheral, α-helical regions are joined by a handlelike structure consisting of two symmetrical, antiparallel β-sheets (Fig. 1) (19). Thus, alterations in the Y62–K70 segment can lead to distortions in the handlelike structure that may be unfavorable for *S. cerevisiae*’s allocation of the α-helix-containing parts of Rps26. This might induce structural disturbance within the yeast ribosome that could affect the productive interaction of its components during protein synthesis. Interestingly, the α-helix-containing regions displayed stronger conservation in yeast, human, and archaeal proteins than did the handlelike structure, which includes the 62-YALPKTYNK-70 motif of the yeast protein. In the archaeon *Staphylothermus marinus*, the latter segment is even truncated (Fig. 1B), and Rps26 from *S. marinus* does not complement the growth of an *S. cerevisiae* Δ*rps26a* Δ*rps26b* strain (Y. Belyi, A. Belyy, and I. Tabakova, unpublished data).

The molecular mechanism by which Rps26 affects the assembly of 80S ribosomes is currently not understood. One possible role of Rps26 in protein synthesis seems to be linked to its interaction with initiation factor eIF3 (1), which is composed of 13 (human) or 6 (yeast) subunits (34). During initiation of translation, eIF3 performs important scaffolding functions for different proteins that assemble on the 40S subunit (35). Importantly, binding of the eIF3 complex to the 40S subunit involves interaction of the so called “left arm” of eIF3 with Rps26 and Rps1 (35). Therefore, structural alterations within Rps26 may influence eIF3 binding and affect 80S ribosome assembly during the initiation of protein synthesis.

Our experiments revealed a shortage of the 40S subunit in yeast strains expressing Rps26^5A^ or Rps26-Hs. This was likely due to specific degradation of small subunits containing mutated or heterologous versions of Rps26. Pathways directed to degradation of defective ribosomes and ribosomal subunits, like ribophagy (36–38) or rRNA decay (39, 40), are only beginning to emerge. Interestingly, a direct link between mutations within ribosomal proteins of the small subunit and autophagy was recently demonstrated (41). By what mechanism the number of small ribosomal subunits in yeast strains expressing mutants of Rps26 is reduced awaits further investigation.

## MATERIALS AND METHODS

### Strains, vectors, and culture conditions.

Cloning was performed in *Escherichia coli* strain DH10B (Invitrogen). Genomic DNA from *S. cerevisiae* strain D273-10B (42) was used for the amplification of *TRP1* and *LEU2* marker genes. *S. cerevisiae* strain MH272-3fα (*ura3 leu2 his3 trp1 ade2*) and the diploid strain MH272-3fα/a (*ura3*/*ura3 leu2*/*leu2 his3*/*his3 trp1*/*trp1 ade2*/*ade2*) (43) are the wild-type yeast strains used to engineer all mutant strains used in this study. The plasmids used for cloning of deletion cassettes were based on pUC19 (New England Biolabs, Frankfurt am Main, Germany). Yeast expression plasmids were constructed using pRS313 (44), pYCplac33, pYEplac195 (45), pYEplac555 (46), YEpTef555 (47), and pESC-Ura (Stratagene). Strains, plasmids, and PCR primers are detailed in Tables S1 to S4 in the supplemental material.

10.1128/mSphere.00109-15.8 Table S2  Plasmids used in the current study. Download Table S2, DOCX file, 0.1 MB. Copyright © 2016 Belyy et al. 2016 Belyy et al. This content is distributed under the terms of the Creative Commons Attribution 4.0 International license.

10.1128/mSphere.00109-15.9 Table S3  Primers used for gene cloning/mutagenesis in the current study. Download Table S3, DOCX file, 0.1 MB. Copyright © 2016 Belyy et al. 2016 Belyy et al. This content is distributed under the terms of the Creative Commons Attribution 4.0 International license.

10.1128/mSphere.00109-15.10 Table S4  Primers used in PCR analysis of *S. cerevisiae* deletion mutants. Download Table S4, DOCX file, 0.1 MB. Copyright © 2016 Belyy et al. 2016 Belyy et al. This content is distributed under the terms of the Creative Commons Attribution 4.0 International license.

*E. coli* strains were grown in LB medium supplemented with the appropriate antibiotic. Yeast strains were grown on rich medium (1% yeast extract, 2% peptone, 2% glucose [YPD]) or on minimal medium containing 0.67% yeast nitrogen base without amino acids (Difco; Becton, Dickinson and Co., Franklin Lakes, NJ) with 2% glucose (SDex) or 2% galactose (SGal). SDex and SGal media were supplemented with the appropriate additives (i.e., uracil, leucine, histidine, tryptophan, or/and adenine).

### Construction of Δ*rps26* deletion strains.

The functional *LEU2* and *TRP1* marker genes for *RPS26* gene disruptions were amplified with their own promoters and terminators from the *S. cerevisiae* D273-10B genomic DNA. Mutations *rps26a*::*LEU2* (Δ*rps26a*) and *rps26b*::*TRP1* (Δ*rps26b*) were constructed by replacing nucleotides 19 to 324 of *RPS26a* or *RPS26b* with the functional *LEU2* or *TRP1* gene cassette, respectively. Yeast transformations were performed by the lithium acetate method (48). Since the Δ*rps26a* Δ*rps26b* double deletion is lethal (11), the diploid *S. cerevisiae* strain SC222 (*RPS26a/rps26a*::*LEU2 RPS26b/rps26b*::*TRP1*) (a full list of engineered strains is shown in Table S1 in the supplemental material) was transformed with YCplac33-*RPS26a* under the control of the *TEF1* promoter (plasmid p887; a full list of engineered plasmids is shown in Table S2). The resulting strain (SC246) was subsequently sporulated and dissected (dissecting microscope MSM manual; Singer Instruments, Somerset, United Kingdom). After tetrad analysis, a haploid *S. cerevisiae* (Δ*rps26a*/Δ*rps26b*+YCplac33-*RPS26a*) isolate (SC254) was selected for subsequent experiments. YCplac33-*RPS26a* was replaced by pRS313-based plasmids encoding different Rps26 variants via the 5-fluoroorotic acid (5-FOA) method (25).

### Cloning and mutagenesis of the *RPS26* genes.

For expression in yeast, *RPS26a* was cloned with its own promoter (pRS313-based plasmid p892) or with the *TEF1* promoter (pRS313-based plasmid p896 and YCplac33-based plasmid p887).

For site-directed mutagenesis, *RPS26a* was amplified with primers #721 and #725 (a full list of primers used for PCR is shown in Table S3 in the supplemental material) and cloned into pUC19 (plasmid p861). The resulting plasmid was used as a template to generate mutations via the QuikChange method (49) as suggested by the manufacturer (Agilent Technologies, Waldbronn, Germany). The Rps26 mutant with five alanine substitutions (Rps26^5A^) and the Rps26^del9^ variant lacking 9 amino acid residues (62-YALPKTYNK-70) were generated by the PCR splicing method (50). All mutated genes were subcloned into YEpTef555 (47) and were then transferred *en bloc* with the upstream *TEF1* promoter into pRS313 using EcoRI/SalI restriction endonuclease sites.

The human gene coding for Rps26-Hs (pET-15-rps26e) was a generous gift from G. Karpova (Novosibirsk Institute for Bioorganic Chemistry, Russia) (9). The coding sequence of Rps26-Hs was placed under the control of the *TEF1* promoter and was cloned into pRS313 (plasmid p1369). Constitutively expressed *RPS26a* and *rps26^del9^* with a COOH-terminal c*-myc* tag were constructed in pESC-His, containing the *TEF1* promoter instead of the original *GAL1/10* promoter, by exchanging the stop codon for a cysteine codon. Subsequently, the fragments were transferred, together with the *TEF1* promoter, into the low-copy-number vector pRS313 using EcoRI/ClaI restriction endonuclease sites (plasmids p1687 and p1692).

### Growth phenotype assay.

Mutant strains were analyzed on agar plates by the drop test. To that end, 5-fold serial dilutions of overnight agar cultures adjusted to the same optical density at 595 nm (OD_595_) were spotted onto YPD, SDex, or SGal plates containing the required supplements. The plates were incubated for the times and temperatures indicated in the legends to the figures in which the results of the experiments are shown.

### Ultracentrifugation studies.

Yeast strains were grown overnight in liquid medium to an OD_600_ of 0.8 to 1.0, quickly chilled on ice, and supplemented with 0.1-mg/ml cycloheximide. Cells were collected by centrifugation at 4,000 rpm for 5 min, washed once with the lysis buffer (20 mM HEPES-K, pH 7.4, 120 mM KCl, 2 mM MgCl_2_, 2 mM DTT, 0.1-mg/ml cycloheximide), and transferred into 2-ml tubes. Extracts were prepared by vortexing yeast cell suspensions with glass beads in the presence of protease inhibitor cocktail (F. Hoffmann-La Roche Ltd., Basel, Switzerland) and 1 mM phenylmethylsulfonyl fluoride (PMSF) using a FastPrep-24 device (MP Biomedicals, Santa Ana, CA). Extracts were clarified by centrifugation at 8,000 rpm for 10 min, followed by centrifugation at 13,500 rpm for 15 min at 4°C (43).

For ribosome sedimentation experiments, clarified cell extracts (two *A*_260_ units in 60 µl) were loaded on top of 90 µl of a 25% sucrose cushion in the lysis buffer and were subjected to ultracentrifugation in a CS 150NX micro-ultracentrifuge (Hitachi, Japan) equipped with an S100AT3 rotor at 95,000 rpm for 35 min (43). The supernatant and the pellet, which contained ribosomal particles, were analyzed via Western blotting with specific antisera as indicated in the legends to the figures in which the results of the experiments are shown.

For polysome profile analysis, clarified supernatants (10 *A*_260_ units) were loaded on top of an 11-ml linear 15-to-55% sucrose gradient in the lysis buffer and subjected to ultracentrifugation for 2.5 h at 39,000 rpm (Sorvall TH641 rotor; Thermo Fisher Scientific, Waltham, MA). Fractions were collected from top to bottom with a density gradient fractionator monitored at *A*_254_ (Teledyne Isco, Lincoln, NE) and were subsequently analyzed by Western blotting using specific antisera as indicated in the legends to the figures in which the results of the experiments are shown.

### rRNA analysis.

*S. cerevisiae* strains were cultivated in SDex liquid medium to an OD_600_ of 1.0, collected by centrifugation, and washed once with distilled water and were then resuspended in 0.5 M NaCl, 10 mM EDTA, 1% SDS, 0.2 M Tris-HCl, pH 7.4. Cells were lysed by glass beads and phenol-chloroform-isoamyl alcohol treatment. Total RNA was ethanol precipitated and analyzed by 1.3% agarose–TBE gel electrophoresis. Prior to electrophoresis, samples (4 to 5 µg of RNA) were mixed with 80% *N*,*N*-dimethylformamide and heated at 65°C for 15 min (51, 52).

### General biochemical methods.

Yeast extracts were analyzed by polyacrylamide gel electrophoresis in sodium dodecyl sulfate buffer (53) followed by Western blotting (54). Crude yeast extracts for Western blot analysis were prepared by the sodium hydroxide method (55). For protein immunodetection experiments, the following antibody and antisera were used: anti-*myc*–horseradish peroxidase (HRP) antibody (catalog no. R951-25; Life Technologies, Moscow, Russia), yeast anti-Rpl24 antiserum, anti-Sse1 antiserum, and anti-Rps9 antiserum (56).
