# Supplementary material for: Ribosomal Protein Rps26 Influences 80S Ribosome Assembly in Saccharomyces cerevisiae
Source: mSphere. 2016 Feb 24;1(1):e00109-15. doi: 10.1128/mSphere.00109-15 (PMC4863615; doi:10.1128/mSphere.00109-15)
Supplement: Text S1 [file sph001162032s1.docx]

**Supplemental Text S1**

**Construction of *S. cerevisiae* Δ*rps26a*Δ*rps26b* strains.** First we engineered diploid yeast strains containing large deletions within the coding sequences of *RPS26a* or *RPS26b*. To that end, 305 out of 360 nucleotides of the coding sequences of *RPS26a* or *RPS26b*, respectively were substituted with the *LEU2* or *TRP1* marker genes, inactivating one allele of the corresponding gene in the diploid strain. Correct insertion of the marker genes was confirmed by PCR with primers annealing outside of the recombination region (Supplementary Fig. S4, Table S4). Dissection of the resulting diploid [*RPS26a/rps26a::LEU2*, *RPS26b/rps26b::TRP1*] strain (SC222, see Supplemental table S1) confirmed lethality of the *rps26a*Δ*rps26b* double deletion (Supplementary Fig. S5). To rescue the lethal phenotype, SC222 was transformed with the plasmid, containing *RPS26a* under the control of the constitutive *TEF1* promoter (plasmid p887). The resulting yeast strain SC246 was then subjected to sporulation and dissection. Tetrad analysis allowed selection of a strain [*rps26a::LEU2*, *rps26b::TRP1*] + *pRPS26a* [*URA3*], which was employed for further studies (SC254).

**References for Supplemental Material**

1. **Peisker K, Braun D, Wolfle T, Hentschel J, Funfschilling U, Fischer G, Sickmann A, Rospert S.** 2008. Ribosome-associated complex binds to ribosomes in close proximity of Rpl31 at the exit of the polypeptide tunnel in yeast. Mol Biol Cell **19:**5279-5288.

2. **Sherman F.** 1964. Mutants of yeast deficient in cytochrome C. Genetics **49:**39-48.

3. **Sikorski RS, Hieter P.** 1989. A system of shuttle vectors and yeast host strains designed for efficient manipulation of DNA in *Saccharomyces cerevisiae*. Genetics **122:**19-27.

4. **Gietz RD, Sugino A.** 1988. New yeast-*Escherichia coli* shuttle vectors constructed with in vitro mutagenized yeast genes lacking six-base pair restriction sites. Gene **74:**527-534.

5. **Conz C, Otto H, Peisker K, Gautschi M, Wolfle T, Mayer MP, Rospert S.** 2007. Functional characterization of the atypical Hsp70 subunit of yeast ribosome-associated complex. J Biol Chem **282:**33977-33984.

6. **Belyi Y, Tartakovskaya D, Tais A, Fitzke E, Tzivelekidis T, Jank T, Rospert S, Aktories K.** 2012. Elongation factor 1A is the target of growth inhibition in yeast caused by *Legionella pneumophila* glucosyltransferase Lgt1. J Biol Chem **287:**26029-26037.

7. **Malygin A, Baranovskaya O, Ivanov A, Karpova G.** 2003. Expression and purification of human ribosomal proteins S3, S5, S10, S19, and S26. Protein Expression and Purification **28:**57-62.

8. **Schneider CA, Rasband WS, Eliceiri KW.** 2012. NIH Image to ImageJ: 25 years of image analysis. Nat Methods **9:**671-675.

9. **Steffen KK, McCormick Ma, Pham KM, MacKay VL, Delaney JR, Murakami CJ, Kaeberlein M, Kennedy BK.** 2012. Ribosome deficiency protects against ER stress in *Saccharomyces cerevisiae*. Genetics **191:**107-118.
