# Supplementary material for: Ribosomal Protein Rps26 Influences 80S Ribosome Assembly in Saccharomyces cerevisiae
Source: mSphere. 2016 Feb 24;1(1):e00109-15. doi: 10.1128/mSphere.00109-15 (PMC4863615; doi:10.1128/mSphere.00109-15)
Supplement: Table S1 [file sph001162032st7.docx]

Table S1. Strains used in the current study.

| Strain/plasmid | Description/genotype | Source/citation |
| --- | --- | --- |
| *E. coli* DH10B | F– *mcr*A Δ(*mrr*-*hsd*RMS-*mcr*BC) Φ80*lac*ZΔM15 Δ*lac*X74 *rec*A1 *end*A1 *ara*D139 Δ(*ara leu*) 7697 *gal*U *gal*K *rps*L *nup*G λ– | Invitrogen |
| *S. cerevisiae* MH272-3fa/α | *ura3/ura3, leu2/leu2, his3/his3, trp1/trp1, ade2/ade2* | (1) |
| *S. cerevisiae* MH272-3fα | *ura3, leu2, his3, trp1, ade2* | (1) |
| *S. cerevisiae* D273-10B | *MAT*α *mal [rho+]* | (2) |
| *S. cerevisiae* SC222 | *RPS26a/rps26a::LEU2, RPS26b/rps26b::TRP1* | This study |
| *S. cerevisiae* SC246 | [*RPS26a/rps26a::LEU2, RPS26b/rps26b::TRP1*] + RPS26 (p887)*[URA3]* | This study |
| *S. cerevisiae* SC254 | [*rps26a::LEU2, rps26b::TRP1*] + RPS26 (p887)*[URA3]* | This study |
| *S. cerevisiae* SC288 | [*rps26a::LEU2, rps26b::TRP1*] + *RPS26* (p892)*[HIS3]* | This study |
| *S. cerevisiae* SC289 | [*rps26a::LEU2, rps26b::TRP1*] + *RPS26 L64A (*p937)*[HIS3]* | This study |
| *S. cerevisiae* SC290 | [*rps26a::LEU2, rps26b::TRP1*] + *RPS26 Y62A* (p938)*[HIS3]* | This study |
| *S. cerevisiae* SC306 | [*rps26a::LEU2, rps26b::TRP1* + *RPS26* (p896)*[HIS3]* | This study |
| *S. cerevisiae* SC307 | [*rps26a::LEU2, rps26b::TRP1*] + *RPS26 Y68A* (p949)*[HIS3]* | This study |
| *S. cerevisiae* SC308 | [*rps26a::LEU2, rps26b::TRP1*] + *RPS26 P65A* (p968)*[HIS3]* | This study |
| *S. cerevisiae* SC309 | [*rps26a::LEU2, rps26b::TRP1*] + *RPS26 K66A* (p969)*[HIS3]* | This study |
| *S. cerevisiae* SC310 | [*rps26a::LEU2, rps26b::TRP1*] + *RPS26 T67A* (p970)*[HIS3]* | This study |
| *S. cerevisiae* SC319 | [*rps26a::LEU2, rps26b::TRP1*] + *RPS26 N69A* (p955)*[HIS3]* | This study |
| *S. cerevisiae* SC320 | [*rps26a::LEU2, rps26b::TRP1*] + *RPS26 K70A* (p974)*[HIS3]* | This study |
| *S. cerevisiae* SC321 | [*rps26a::LEU2, rps26b::TRP1*] + *RPS26 L71A* (p951)*[HIS3]* | This study |
| *S. cerevisiae* SC349 | [*rps26a::LEU2, rps26b::TRP1*] + *RPS26^5A^* (p1032)*[HIS3]* | This study |
| *S. cerevisiae* SC482 | [*rps26a::LEU2, rps26b::TRP1*] + RPS26 (p887)*[URA3]* + *RPS26^del9^* (p1246)*[HIS3]* | This study |
| *S. cerevisiae* SC511 | [*ura3, leu2, his3, trp1, ade2*] + pRS313*[HIS3]* | This study |
| *S. cerevisiae* SC512 | [*ura3, leu2, his3, trp1, ade2*] + RPS26 (p896)*[HIS3]* | This study |
| *S. cerevisiae* SC513 | [*ura3, leu2, his3, trp1, ade2*] + RPS26^del9^ (p1246)*[HIS3]* | This study |
| *S. cerevisiae* SC557 | [*rps26a::LEU2*, */rps26b::TRP1*] + *RPS26-Hs* (p1369)*[HIS3]* | This study |
| *S. cerevisiae* SC697 | *rps26a::LEU2*, */rps26b::TRP1* + RPS26 (p887)*[URA3]* + *RPS26[-9aa]::c-myc* (p1692)*[HIS]* | This study |
| *S. cerevisiae* SC699 | *rps26a::LEU2*, */rps26b::TRP1* + *RPS26::c-myc* (p1687)*[HIS]* | This study |
