# Supplementary figures and images for: Ribosomal Protein Rps26 Influences 80S Ribosome Assembly in Saccharomyces cerevisiae
Source: mSphere. 2016 Feb 24;1(1):e00109-15. doi: 10.1128/mSphere.00109-15 (PMC4863615; doi:10.1128/mSphere.00109-15)

Figure S1.

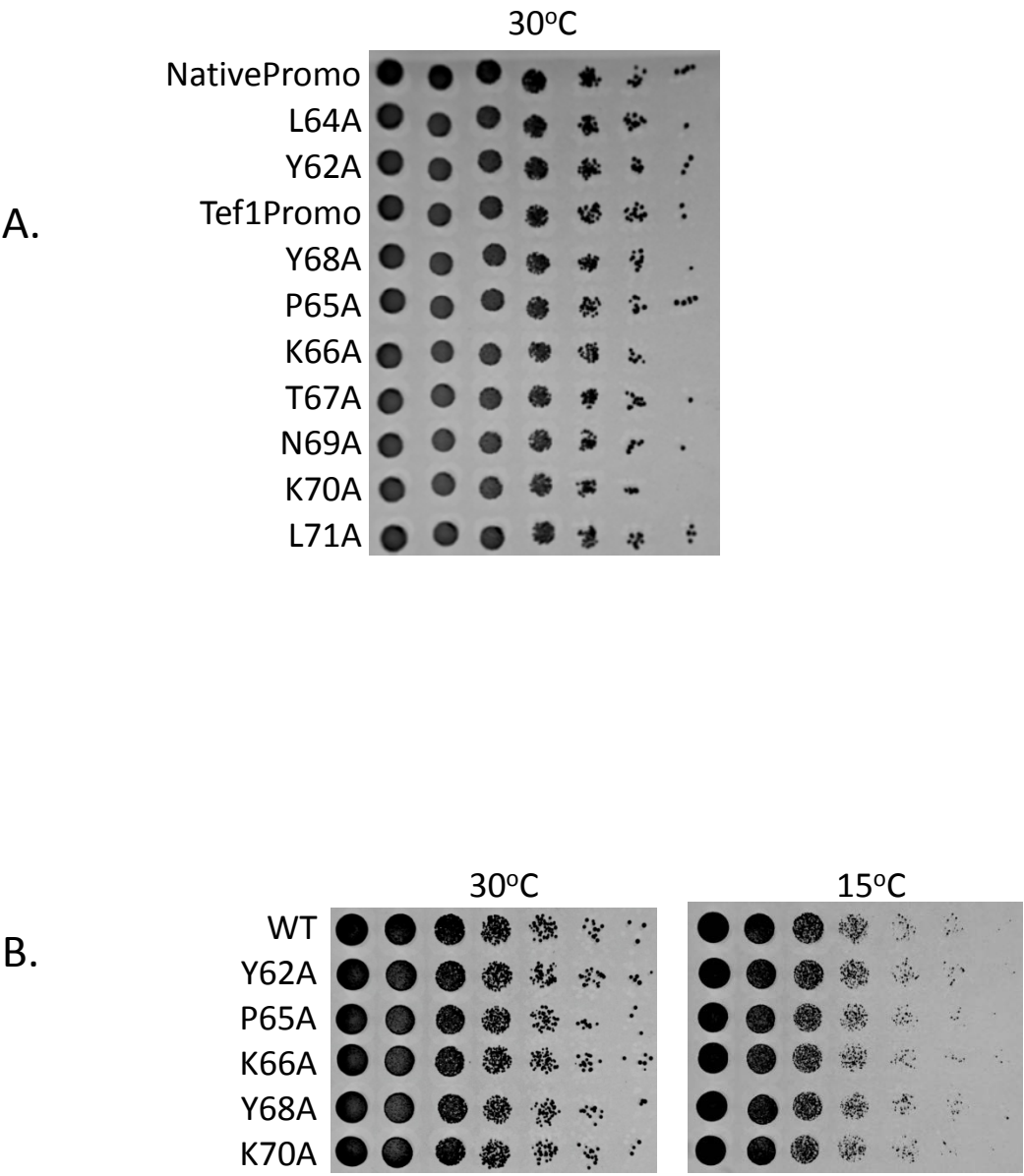

Supplement: Figure S1 [file sph001162032sf2.pdf]

Figure S2.

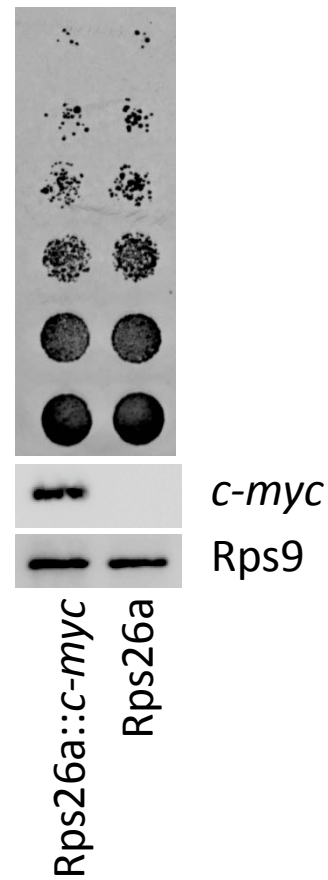

Supplement: Figure S2 [file sph001162032sf3.pdf]

Figure S3.

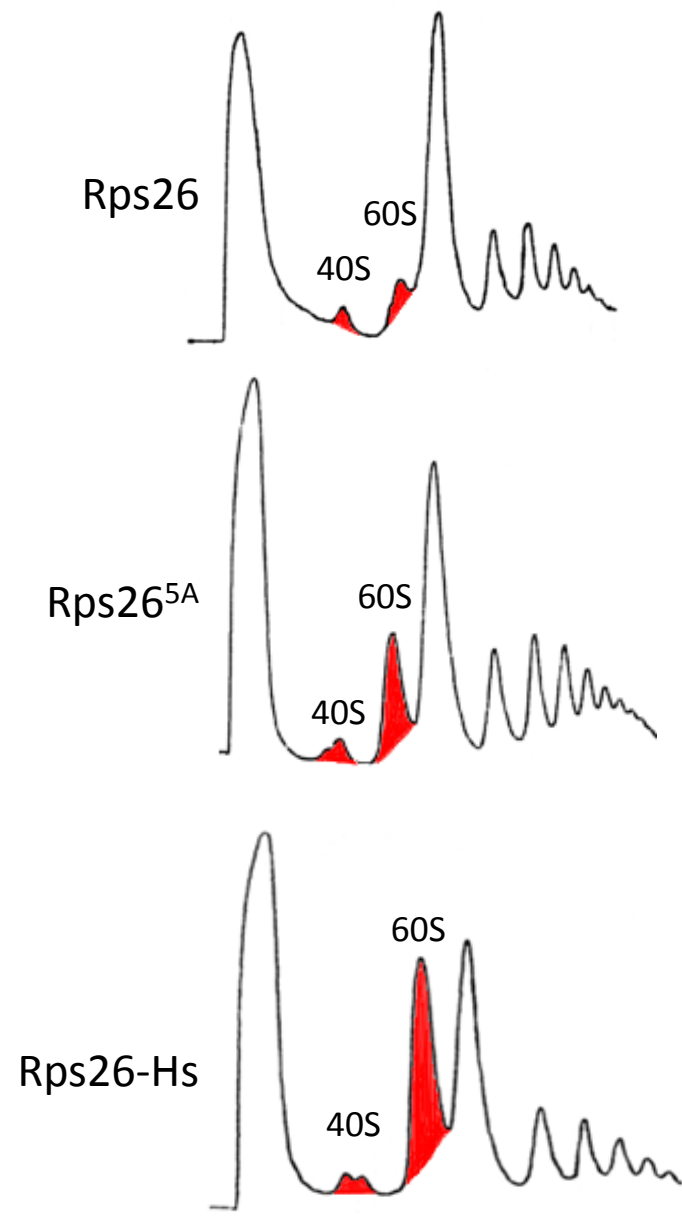

Supplement: Figure S3 [file sph001162032sf4.pdf]

Figure S4.

A.

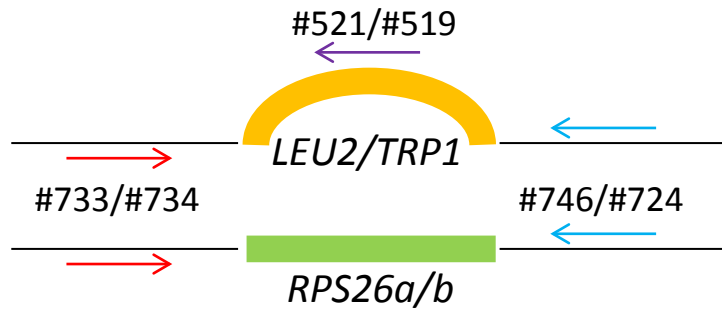

B.

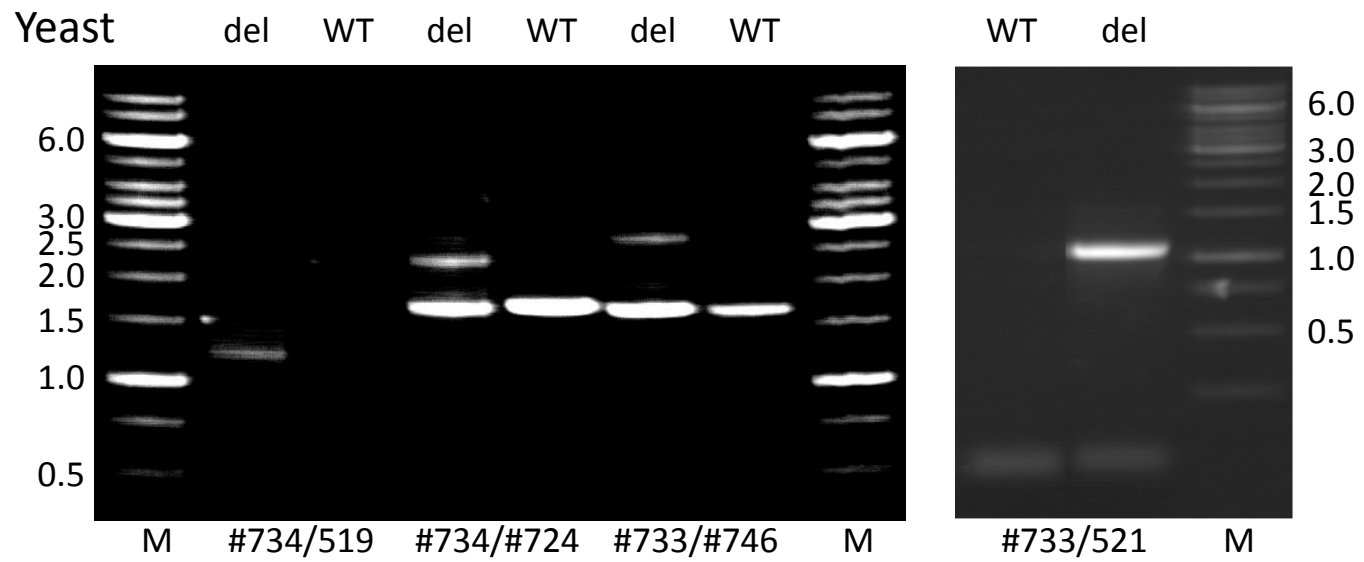

Supplement: Figure S4 [file sph001162032sf5.pdf]

Figure S5.

A.

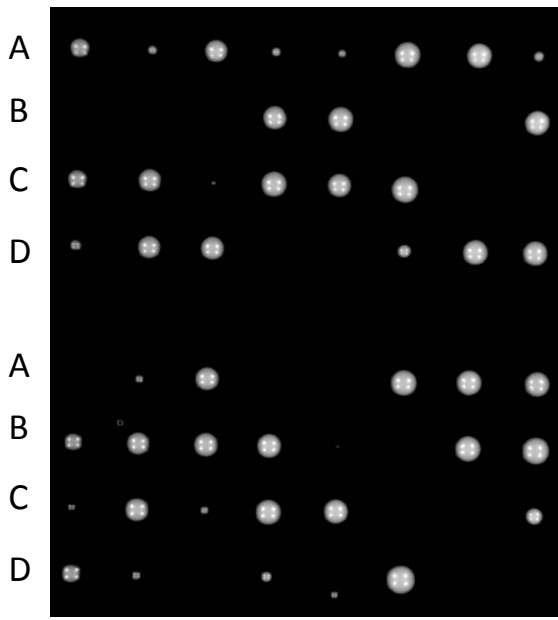

B.

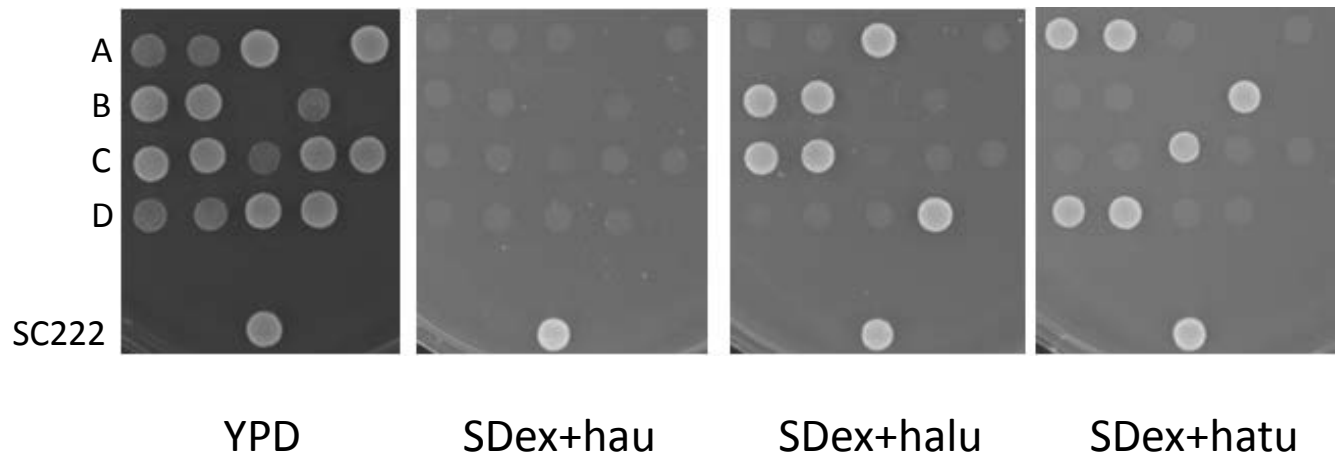

Supplement: Figure S5 [file sph001162032sf6.pdf]
