# Supplementary material for: Ribosomal Protein Rps26 Influences 80S Ribosome Assembly in Saccharomyces cerevisiae
Source: mSphere. 2016 Feb 24;1(1):e00109-15. doi: 10.1128/mSphere.00109-15 (PMC4863615; doi:10.1128/mSphere.00109-15)
Supplement: Table S2 [file sph001162032st8.docx]

Table S2. Plasmids used in the current study

| Plasmid/vector | Description | Source/citation |
| --- | --- | --- |
| pUC19 | *E. coli g*eneral cloning vector (Ap) | NEB |
| pRS313 | *E. coli*/*S. cerevisiae* shuttle vector [HIS3] | (3) |
| pESC-Ura | *E. coli*/*S. cerevisiae* shuttle vector [URA3] | Stratagene |
| YEplac195 | *E. coli*/*S. cerevisiae* shuttle vector [URA3] | (4) |
| YEplac555 | *E. coli*/*S. cerevisiae* shuttle vector [ADE2] | (5) |
| YCplac33 | *E. coli*/*S. cerevisiae* shuttle vector [URA3] | (4) |
| YEpTEF555 | *TEF1* promoter in YEplac555 | (6) |
| p967 | Human *RPS26e* in pET15b | (7) |
| p847 | *TEF1* promoter::*RPS26a* in YCplac555 | This study |
| p861 | *RPS26* in pUC19 | This study |
| p865 | *RPS26a*-deleting cassette in pUC19 [LEU2] | This study |
| p866 | *RPS26b*-deleting cassette in pUC19 [TRP1] | This study |
| p887 | *TEF1* promoter::*RPS26a* in YCplac33 | This study |
| p892 | *RPS26a* with its own promoter in pRS313 | This study |
| p896 | *TEF1* promoter::*RPS26a* in pRS313 | This study |
| p937 | *TEF1* promoter::*RPS26a* L64A in pRS313 | This study |
| p938 | *TEF1* promoter::*RPS26a* Y62A in pRS313 | This study |
| p949 | *TEF1* promoter::*RPS26a* Y68A in pRS313 | This study |
| p951 | *TEF1* promoter::*RPS26a* L71A in pRS313 | This study |
| p955 | *TEF1* promoter::*RPS26a* N69A in pRS313 | This study |
| p968 | *TEF1* promoter::*RPS26a* P65A in pRS313 | This study |
| p969 | *TEF1* promoter::*RPS26a* K66A in pRS313 | This study |
| p970 | *TEF1* promoter::*RPS26a* T67A in pRS313 | This study |
| p974 | *TEF1* promoter::*RPS26a* K70A in pRS313 | This study |
| p1032 | *TEF1* promoter::*RPS26a*^5A^ in pRS313 | This study |
| p1246 | *TEF1* promoter::*RPS26^del9^* in pRS313 | This study |
| p1369 | *TEF1* promoter::*RPS26-Hs* in pRS313 | This study |
| p1687 | *TEF1* promoter:: *Rps26a::c-myc* in pRS313 | This study |
| p1692 | *TEF1* promoter:: *Rps26^del9^::c-myc* in pRS313 | This study |
