# Supplementary material for: Ribosomal Protein Rps26 Influences 80S Ribosome Assembly in Saccharomyces cerevisiae
Source: mSphere. 2016 Feb 24;1(1):e00109-15. doi: 10.1128/mSphere.00109-15 (PMC4863615; doi:10.1128/mSphere.00109-15)
Supplement: Table S3 [file sph001162032st9.docx]

Table S3. Primers used for gene cloning/mutagenesis in the current study

| ID number | Nucleotide sequence (5’-to-3’) | Resulting plasmid |
| --- | --- | --- |
| #357 | CGTCGTCATCCTTGTAATCCATCG | p1692, p1687 |
| #721 | TTAGTTAAGGATCT**GTCGAC**GTGCGC | p847, p887, p892, p896 |
| #725 | ATTAGA**GGATCC**AAAATGCCAAAG | p847, p887, p896 |
| #728 | TATCTACCAACGATTTGAC | p1369 |
| #765 | CTTA**GAATTC**AAGTTGCCCTTCTG | p892 |
| #770 | CCTGAAGCCGCTTTGCCAAAGACT | p938 |
| #771 | AGTCTTTGGCAAAGCGGCTTCAGG | p938 |
| #772 | GAATACGCTGCGCCAAAGACTTAC | p937 |
| #773 | GTAAGTCTTTGGCGCAGCGTATTC | p937 |
| #776 | TTGCCAGCGACTTACAACAAGTTA | p969 |
| #777 | TAACTTGTTGTAAGTCGCTGGCAA | p969 |
| #778 | CCAAAGGCCTACAACAAGTTACAC | p970 |
| #779 | GTGTAACTTGTTGTAGGCCTTTGG | p970 |
| #780 | CCAAAGACTGCCAACAAGTTACA | p949 |
| #781 | TGTAACTTGTTGGCAGTCTTTGG | p949 |
| #782 | ACATATGCCAAGTTACACTACTGTG | p955 |
| #783 | CACAGTAGTGTAACTTGGCATATGT | p955 |
| #784 | TTACAACGCGTTACACTACTGTGT | p974 |
| #785 | ACACAGTAGTGTAACGCGTTGTAA | p974 |
| #786 | AACAAGGCACACTACTGTGTTTCT | p951 |
| #787 | AGAAACACAGTAGTGTGCCTTGTT | p951 |
| #796 | CACAGTAGTGTAACTTGGCATATGT | p968 |
| #797 | GTAAGTCTTTGCCAAAGCGTATTC | p968 |
| #814 | GCCGCTTTGGCAGCGACTGCCAACGCGTTACACTAC | p1032 |
| #815 | GTAGTGTAACGCGTTGGCAGTCGCTGCCAAAGCGGC | p1032 |
| #958 | GTAGTGTAATTCAGGGTAGACAGAAGCCTCGGA | p1246 |
| #959 | TACCCTGAATTACACTACTGTGTTTCTTGTGCT | p1246 |
| #1043 | CGCA**ACTAGT**CAGTGAGCGAGG | p1369 |
| #1311 | TCAGTG**ATCGAT**GAAGCGGAAGA | p1692, p1687 |

Used restriction endonuclease sites are shown in bold and underlined font
