# Supplementary material for: Ribosomal Protein Rps26 Influences 80S Ribosome Assembly in Saccharomyces cerevisiae
Source: mSphere. 2016 Feb 24;1(1):e00109-15. doi: 10.1128/mSphere.00109-15 (PMC4863615; doi:10.1128/mSphere.00109-15)
Supplement: Table S4 [file sph001162032st10.docx]

Table S4. Primers used in PCR analysis of *S. cerevisiae* deletion mutants

| ID number | Nucleotide sequence (5’-to-3’) | Target gene |
| --- | --- | --- |
| #733 | TCCATACACCCACCCATAC | 739 bp upstream of *RPS26a* start codon |
| #521 | CGGCTGTGATTTCTTGACC | *LEU2* |
| #721 | TTAGTTAAGGATCTGTCGACGTGCGC | 424 bp downstream from *RPS26a* stop codon |
| #734 | GAGTAAAGGAGATGGCTTG | 929 bp upstream of *RPS26b* start codon |
| #519 | AGACATACTCCAAGCTGCCT | *TRP1* |
| #724 | CCTCGTCGACTGTAATAGTTGATGG | 406 bp downstream from *RPS26b* stop codon |
| #746 | CAATTCTTCCAGTCTCTTCG | 566 bp downstream from *RPS26a* stop codon |
